# Keratoderma and ichthyosis as valuable features for the diagnosis of CEDNIK syndrome

**DOI:** 10.1016/j.jdcr.2023.09.005

**Published:** 2023-09-27

**Authors:** Deyson Lorenzo-Ríos, Amara Guerrero-García, Francisco Colón-Fontánez

**Affiliations:** a Department of Dermatology, School of Medicine, University of Puerto Rico, San Juan, Puerto Rico; b School of Medicine, Universidad Central del Caribe, Bayamón, Puerto Rico

**Keywords:** CEDNIK syndrome, cerebral dysgenesis, ichthyosis, keratoderma

## Introduction

Cerebral dysgenesis, neuropathy, ichthyosis, and keratoderma (CEDNIK) syndrome is a rare autosomal recessive neurocutaneous disorder caused by a loss-of-function mutation in the SNAP29 gene.^1^ This gene encodes synaptosomal-associated protein 29 (SNAP29) required for vesicle trafficking during exocytosis, endocytosis, autophagy, ciliogenesis, and other cellular events.^2^, ^3^, ^4^ Decreased expression of SNAP29 in the skin results in an abnormal maturation of lamellar granules, which are organelles that are most likely found on the upper epidermal layers with the role of properly delivering lipids, proteases, and their inhibitors to the stratum corneum.^1^ Retained glucosylceramide and kallikrein-containing granules in the stratum corneum leads to retention hyperkeratosis and defective barrier formation in patients with CEDNIK syndrome.^1^ The ichthyosiform phenotype due to the abnormal function of lamellar granules has been replicated *in vitro*, reiterating the importance of SNAP29 for normal epidermal differentiation.^5^ Clinical manifestations include severe global developmental delay, hypotonia, neuropathy, microcephaly, facial dysmorphism, and failure to thrive. Cutaneous findings including palmoplantar keratoderma (PPK), and ichthyosis can present either in infancy or have a late onset presentation.^6^^,^^7^ However, the phenotypic description of this syndrome is limited to few cases showing poor prognosis and uniform fatality between ages 5 and 12 years due to the lack of a definite therapy.^1^^,^^2^^,^^5^ Around 22 cases of CEDNIK syndrome have been reported since 2005.^1^^,^^2^^,^^5^^,^^6^^,^^8^ We describe the case of CEDNIK syndrome where patient presented with cutaneous signs that served as clues for the diagnosis.

## Case report

**Fig 1:**
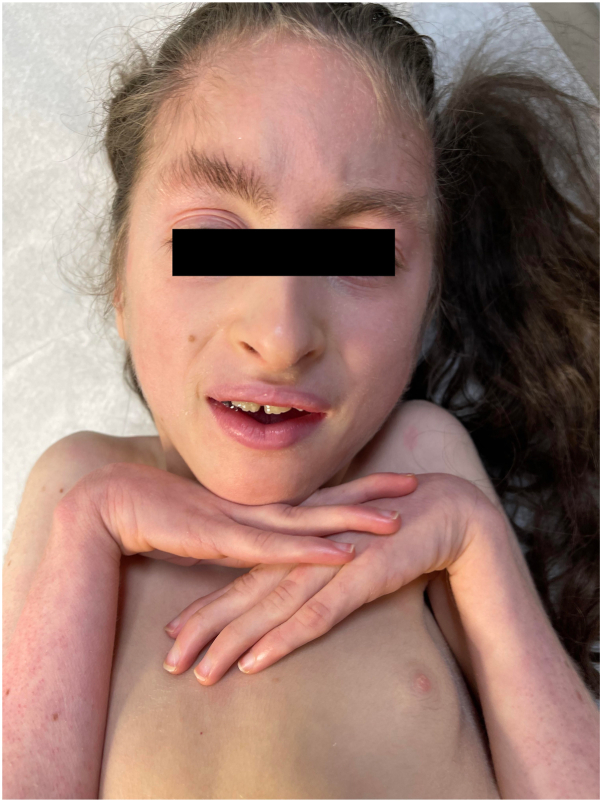
Dysmorphic facies in the patient with CEDNIK syndrome, including microcephaly, bitemporal narrowing, strabismus, thick eyebrows, broad nasal bridge, and short philtrum. *CEDNIK*, cerebral dysgenesis, neuropathy, ichthyosis, and keratoderma.

**Fig 2:**
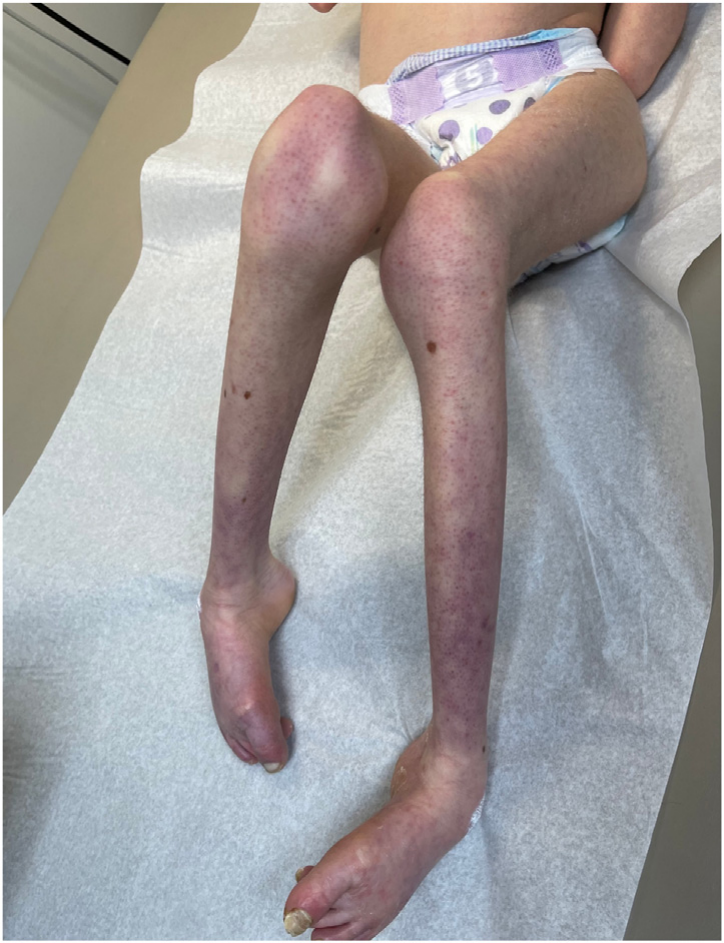
Clubbing of the feet, contractures on distal lower extremity joints and follicular prominence in the patient with CEDNIK syndrome. *CEDNIK*, cerebral dysgenesis, neuropathy, ichthyosis, and keratoderma.

**Fig 3:**
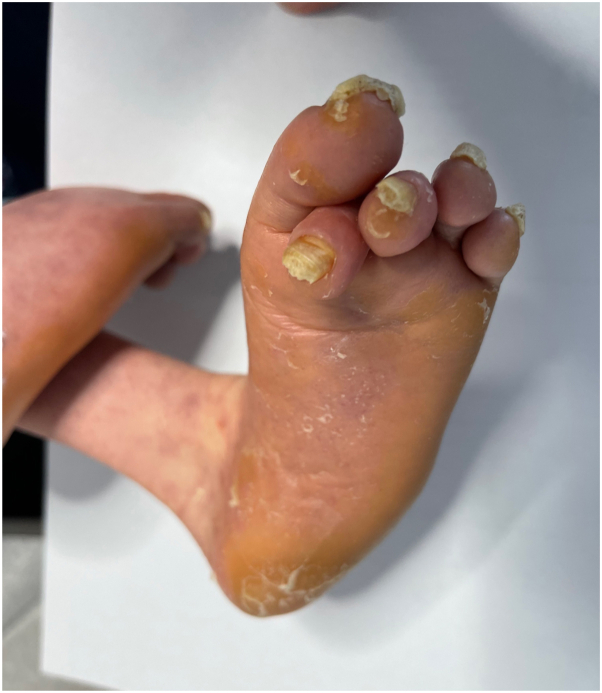
Fine ichthyosiform erythema, dystrophic toenails, and diffuse plantar hyperkeratosis in the patient with CEDNIK syndrome. *CEDNIK*, cerebral dysgenesis, neuropathy, ichthyosis, and keratoderma.

A 13-year-old Hispanic, Puerto Rican female patient with severe developmental delay, microcephaly, and strabismus was brought to clinic due to thick scales on the legs for the last 3 years. The patient has diffuse xerosis and subtle erythema on extremities since birth, both managed with emollients. At age of 10 years the patient developed fine scales on extremities, and progressive thickening of the toenails and soles. Physical examination revealed marked dysmorphic facies (Fig 1), clubbing of the feet and contractures on distal joints, fine ichthyosiform erythema and follicular prominence on arms and legs, and dystrophic toenails (Fig 2). Mucosae and hair were normal. There was prominent diffuse plantar hyperkeratosis (Fig 3) which pointed to the differential diagnosis of ichthyosiform genodermatoses with PPK and neurologic disorders. Brain magnetic resonance imaging were consistent with dysgenesis of cerebral hemispheres and microcephaly. A genetic sequencing panel for PPK and ichthyotic disorders confirmed a homozygous pathogenic variant in the SNAP29 gene, exon 2, c.354dup (p.Leu119Alafs∗15) consistent with autosomal recessive CEDNIK syndrome. A multidisciplinary approach was taken including a neurologist, ophthalmologist, and geneticist to establish a longitudinal plan of care and improve quality of life.

## Discussion

The progressive neuromotor degeneration in our patient was initially attributed to microcephaly. Nonetheless, the patient developed keratoderma and ichthyosis later in life, which prompted suspicion of a genetic neurocutaneous disorder. Cutaneous findings were valuable clues for diagnosing CEDNIK syndrome, which was eventually confirmed with a genetic sequencing as suggested for syndromic ichthyoses with extracutaneous features.^9^

Our patient presented with a variant of the SNAP29 gene, exon 2, c.354dup (p.Leu119Alafs∗15), a sequence change that creates a premature translational stop sign that is expected to result in an absent or disrupted protein product. According to the literature, skin findings such as keratoderma and ichthyosis, neuropathy, and cerebral dysgenesis were initially believed to be sufficient for diagnosing CEDNIK syndrome.^2^ However, in many patients with the pathogenic variants of SNAP29, the absence of skin findings automatically excluded the diagnosis of CEDNIK syndrome, despite the variability in phenotypes that would otherwise support genetic testing as a definite diagnostic tool.^2^

Mah-Som et al^2^ reported an increasing number of patients with SNAP29 pathogenic variants in the literature in which, due to the absence of skin findings such as ichthyosis and keratoderma, the diagnosis of CEDNIK syndrome was not established. Out of 20 patients finally diagnosed with CEDNIK syndrome, 20 (100%) presented with ichthyosis, and 17 (85%) presented with keratoderma, either plantar, palmar, or both.^2^ Our patient developed both ichthyosiform scales and diffuse plantar keratoderma at the first decade of life, consistent with the age of onset in other reported cases of CEDNIK syndrome. PPK and ichthyosis have been reported to occur at different ages in several cases. Fuchs et al^5^ reported 2 cases of CEDNIK syndrome with ichthyosis developed between the first 3 and 7 months of life, whereas PPK was observed around 11 months of life. The keratoderma in our patient was diffuse and limited to soles. Histologic analysis may be helpful for the clinician suspecting this disorder. CEDNIK syndrome-associated ichthyosis may manifest solely with hyperkeratosis, in contrast with other types of ichthyoses, which often feature acanthosis, hyperkeratosis as well as another specific features.^5^

Other clinical manifestations have been described including keratoderma of both palms and soles (PPK), nystagmus, foveal hypoplasia, epicanthal folds, synophrys, long eyelashes, flaring nares, depressed nasal bridge, sensorineural deafness, seizures, hypotonia, areflexia, repetitive behavior, purposeless movements, and others.^2^^,^^5^^,^^7^^,^^8^ Moreover, several imaging anomalies of the central nervous system have been described in CEDNIK syndrome contributing to the phenotypic variation. These includes dysgenesis of the corpus callosum, frontoparietal polymicrogyria, abnormal cortical folding, brainstem malformations, and hypoplastic intraconal optic nerves.^8^

In addition to severe progressive comorbidities and impaired functional status, CEDNIK syndrome carries a poor prognosis, with a lifespan fluctuating between neonatal lethality to 12 years, with the eldest patient reported in the literature reaching 19 years.^2^^,^^10^ A common cause of death mentioned in prior cases of patients with the characteristic features of CEDNIK syndrome is aspiration pneumonia between the ages of 5 and 12 years.^2^

To conclude, keratoderma and ichthyosis served as valuable clues that led to the diagnosis of CEDNIK syndrome in our patient with neurologic findings. A high index of suspicion and correlation between neurologic and skin findings would have prompted an earlier diagnosis. Genetic testing should be considered to confirm the diagnosis given the prognosis implicated in CEDNIK syndrome, phenotypic variation among published cases, and genetic heterogeneity of ichthyosis. Reporting future cases may help to understand the variability of clinical features and provide additional insight to develop a diagnostic criterion for CEDNIK syndrome.

## Conflicts of interest

None disclosed.
